# Reporting Crime Victimizations to the Police and the Incidence of Future Victimizations: A Longitudinal Study

**DOI:** 10.1371/journal.pone.0160072

**Published:** 2016-07-28

**Authors:** Shabbar I. Ranapurwala, Mark T. Berg, Carri Casteel

**Affiliations:** 1 Injury Prevention Research Center, Department of Occupational and Environmental Health, College of Public Health, The University of Iowa, Iowa City, Iowa, United States of America; 2 Department of Sociology, College of Liberal Arts and Science, The University of Iowa, Iowa City, Iowa, United States of America; University of Texas at San Antonio, UNITED STATES

## Abstract

**Background:**

Law enforcement depends on cooperation from the public and crime victims to protect citizens and maintain public safety; however, many crimes are not reported to police because of fear of repercussions or because the crime is considered trivial. It is unclear how police reporting affects the incidence of future victimization.

**Objective:**

To evaluate the association between reporting victimization to police and incident future victimization.

**Methods:**

We conducted a retrospective cohort study using National Crime Victimization Survey 2008–2012 data. Participants were 12+ years old household members who may or may not be victimized, were followed biannually for 3 years, and who completed at least one follow-up survey after their first reported victimization between 2008 and 2012. Crude and adjusted generalized linear mixed regression for survey data with Poisson link were used to compare rates of future victimization.

**Results:**

Out of 18,657 eligible participants, 41% participants reported to their initial victimization to police and had a future victimization rate of 42.8/100 person-years (PY) (95% CI: 40.7, 44.8). The future victimization rate of those who did not report to the police (59%) was 55.0/100 PY (95% CI: 53.0, 57.0). The adjusted rate ratio comparing police reporting to not reporting was 0.78 (95%CI: 0.72, 0.84) for all future victimizations, 0.80 (95% CI: 0.72, 0.90) for interpersonal violence, 0.73 (95% CI: 0.68, 0.78) for thefts, and 0.95 (95% CI: 0.84, 1.07) for burglaries.

**Conclusions:**

Reporting victimization to police is associated with fewer future victimization, underscoring the importance of police reporting in crime prevention. This association may be attributed to police action and victim services provisions resulting from reporting.

## Introduction

Government policies designed to alleviate the public health burden of violence generally consider law enforcement officials as important resources for violence prevention. A fundamental component of such crime-control efforts is the willingness of the public, particularly victims of crime, to mobilize legal authorities to resolve public safety threats [1]. Without active community involvement the police are less likely to detect violent crimes, arrest suspects, and effectively allocate crime-control resources to communities [2]. However, according to recent national-level estimates, approximately 54 percent of violent victimizations are not reported to the police [3].

Several individual, situational, and contextual factors reliably predict whether or not victims mobilize police services [4–7]. Research suggests that reporting of victimizations to police may vary by victim and offender characteristics, including injuries received, presence of bystanders, and use of weapons [8–14]. It is documented that African Americans, Hispanics, and people living in disadvantaged neighborhoods experience higher rates of violence than Whites and those from low-poverty neighborhoods [3,15]. Furthermore, it has been suspected that police services are not as robust or effectively delivered to minority groups in some communities, which could translate into race-specific effects of police notification on subsequent victimization risk [16,17]. Thus, both police notification and its impact on subsequent experiences with violence and property crime may also vary by victim’s racial/ ethnic background.

Victims are also concerned about retaliatory action from perpetrators in case of police notification [18–20] which may preclude them from reporting. Reporting practices are also important for understanding the mechanisms of repeat victimization [21]. Research shows that prior victimization is strongly associated with risk of future victimization [22–25]. Research on repeat victimization raises questions about the implications of police reporting on personal safety. But comparatively few studies have moved beyond the question of “why victims notify the police” to questions about the ramifications of reporting [25,26]. The only study that assessed the personal ramifications of reporting to the police has been conducted in the context of intimate partner violence [25]. Another ecologic study conducted in the United Kingdom assessed the association of anonymous police reporting with violence and violence-related injuries [26]. Both these studies reported that police reporting was associated with fewer future victimization/ crime. But there is no information about how reporting to police affects all forms of interpersonal violence including robberies, assaults, gang violence and property crimes like thefts and burglaries. Addressing this knowledge gap may help inform policies about how police engage victims, particularly those in minority communities.

In this study, we evaluated the association of reporting victimization to police with the incidence of future victimizations. The study used National Crime Victimization Survey (NCVS) data, which is the only nationally representative incident-level crime and victimization data that collects detailed victim and social contextual data about each event [27].

## Methods

### Participants and procedures

We conducted a retrospective cohort study using data from the National Crime Victimization Survey (NCVS) 2008–2012, a nationally representative, self-reported, longitudinal survey of incident non-fatal crime reports, designed to assess the rates of non-fatal crime victimization in the United States (US) [27]. The survey is sponsored by the Bureau of Justice Statistics and the data is collected by the US census bureau. The NCVS is a household based survey of 90,000 households that targets 12 years or older household residents. The housing units and group quarters are clustered within counties, groups of counties, and large metropolitan areas. The survey excludes residents under 12 years of age, crew members of maritime vessels, personnel living in military barracks, the homeless, prison inmates, US citizens living abroad, and foreign visitors [28]. The initial survey is a face-to-face interview after which the participants take telephone surveys every six months for three years. Each participant takes up to seven surveys contributing a maximum of 3.5 years of person-time in the NCVS. Participants who completed at least one follow-up survey after their first reported victimization during 2008–2012 were included in this study. Due to the secondary and de-identified nature of the publicly available National Crime Victimization Survey data used for this study, it was determined ‘not human subject research’ by the Institutional Review Board at the University of Iowa.

### Measures

**Fig 1:**
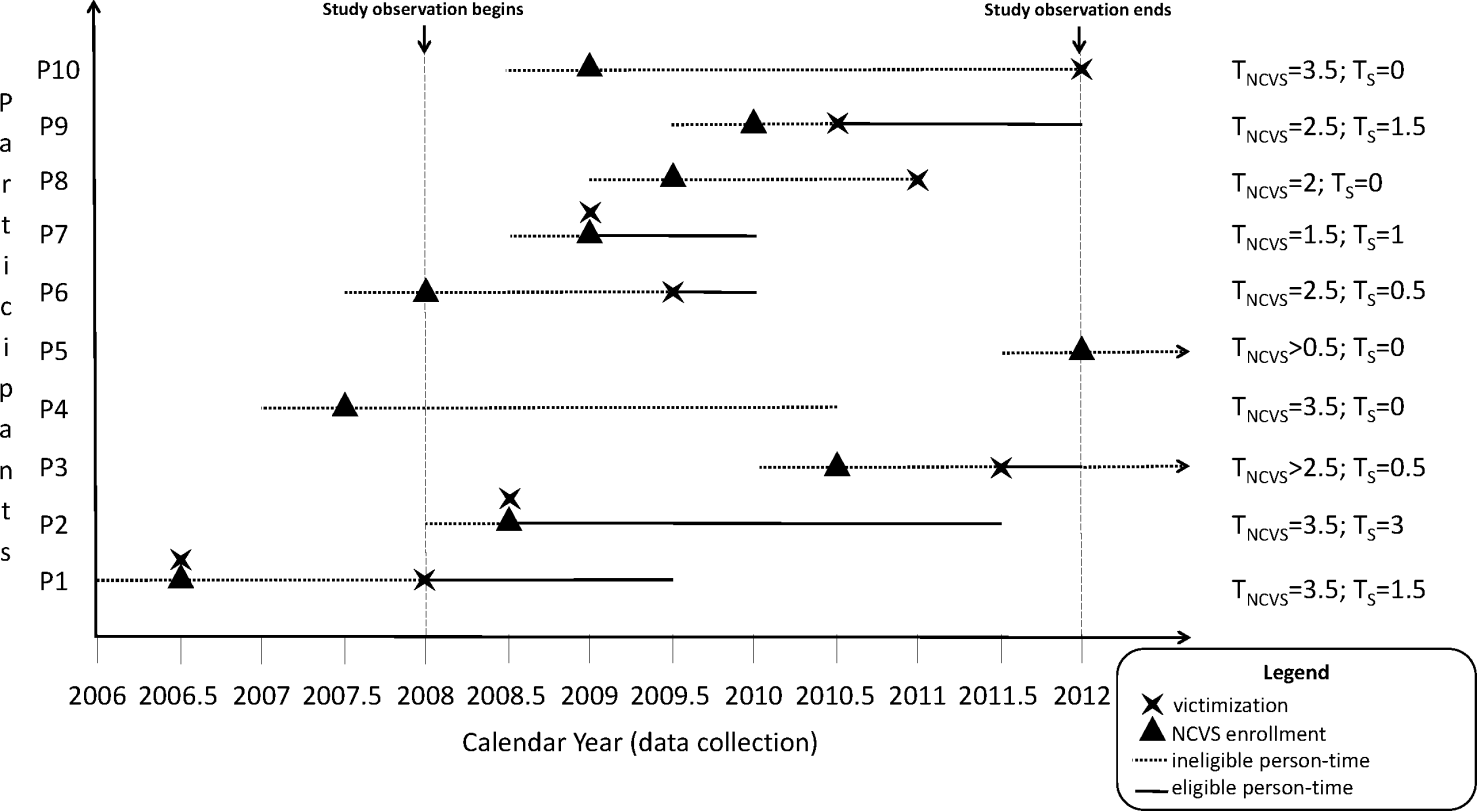
Person-time calculation (in years) for participants P1 through P10 in the NCVS, 2008–2012, US. T_NCVS_, person-time in the NCVS study; T_S_, eligible person-time within the observation period, such that individuals with T_S_ = 0 are ineligible for this study.

Participants who reported their first victimization to the police, between 2008 and 2012, were considered exposed and those who did not report their initial victimization to the police were considered unexposed. Thus the exposure variable was a binary variable. After the initial victimization, they were followed over time to assess the rate of subsequent victimization. The follow-up time varied based on when the participants reported their initial victimization, and their last completed follow-up (Fig 1). Fig 1, provides examples of how the eligible person-time on the study (T_S_) was calculated for each participant and how it compared with the person-time of follow-up on the NCVS study (T_NCVS_). Participants who did not report any victimization (P4), did not take a follow-up survey after the first victimization report (P8), were enrolled towards the end of the follow-up period from July-December 2012 (P5), or reported their first victimization at the end of follow-up period (P10) were not eligible for this study; hence represented in Fig 1 with T_S_ = 0. Participants who were enrolled in NCVS before 2008 (P1 and P4) could have had their first victimization before the one observed in Fig 1, however, we did not have information on that since we defined our study observation period between 2008 and 2012.

During the follow-up surveys participants reported future victimizations that occurred after the initial victimization, these subsequent (or future) victimizations were categorized as interpersonal violence (including sexual assaults, robberies, threatened assaults and threatened rape), burglary (including forced entry into a property), and theft (including motor vehicle theft and pickpocketing). The overall future victimizations, future interpersonal violence victimizations, future burglary victimizations, and future theft victimizations were accounted as count variables. The future victimization counts were divided by the total person time on the study (T_S_) to calculate rates of future victimizations per 100 person-years.

To identify potential covariates for controlling confounding, a directed acyclic graph [29] was developed using previously published literature [4–20] and consensus among the co-authors. A directed acyclic graph, also known as causal diagram, allows to identify a minimal sufficient set of well measured variables that controls for all known confounding. The minimal sufficient set of variables required to control for all known confounding included initial victimization type (interpersonal violence/ burglaries/ thefts), victim’s baseline age (continuous), sex (male/ female), race (White/ African American/ American Indian Alaska Native/ Asian), ethnicity (Hispanic/ non-Hispanic), baseline household income (<$25,000/ $25,000-$49,999/ $50,000 –$74,999/ > = $75,000), education (< high school/ high school graduate/ some college/ associate degree/ bachelor’s degree/ master’s +), offender sex (male/ female/ not known), victim-offender relationship (stranger/ acquaintance), victimization location (inside home/ outside home/ friend’s home/ commercial place/ parking places/ school/ public places/ other), victim injury (yes/ no), and bystander presence (yes/ no/ don’t know). We hypothesized that the relationship between reporting to police and the incidence of future victimization may vary by race of the victim and victim-offender relationship. Hence, we explored effect measure modification by these characteristics by examining the *p*-value of the interaction term in the regression analyses; we reported stratified rate ratio estimates.

### Statistical analysis

We used survey statistics (surveymeans, surveyfreq, surveyreg) in SAS 9.4 (SAS Inc., Cary, NC) to calculate weighted frequencies, rates, and unadjusted and adjusted rate differences of future victimization comparing those who reported their initial victimization to the police and those who did not.

We used generalized linear mixed Poisson models for multilevel survey data to compare the rates of future victimization among those who reported their initial victimization to the police and those who did not, while accounting for clustering in the survey design. These analyses were conducted using SurveyGLIM in LISREL9 (SSI Inc., Skokie, IL) to estimate weighted unadjusted and adjusted rate ratios (RR) and 95% confidence intervals (CI). Statistical analyses were conducted in 2014–2015.

However, the above analyses may have potential selection bias based on when the initial victimization occurred during the follow-up (which is when the exposure to police reporting is measured). This can be assessed by the length of time survey participants were followed after their initial victimization (Fig 1). To assess the effect of the potential selection bias on the association between police reporting and future victimization, we conducted sensitivity analyses. We restricted the analyses sample based on follow-up cutoffs of 6 months, 12 months, and 24 months. We then examined the association of police reporting with future victimization in each of the restricted sample files separately and compared the rate ratios with the overall rate ratio estimate.

## Results

**Fig 2:**
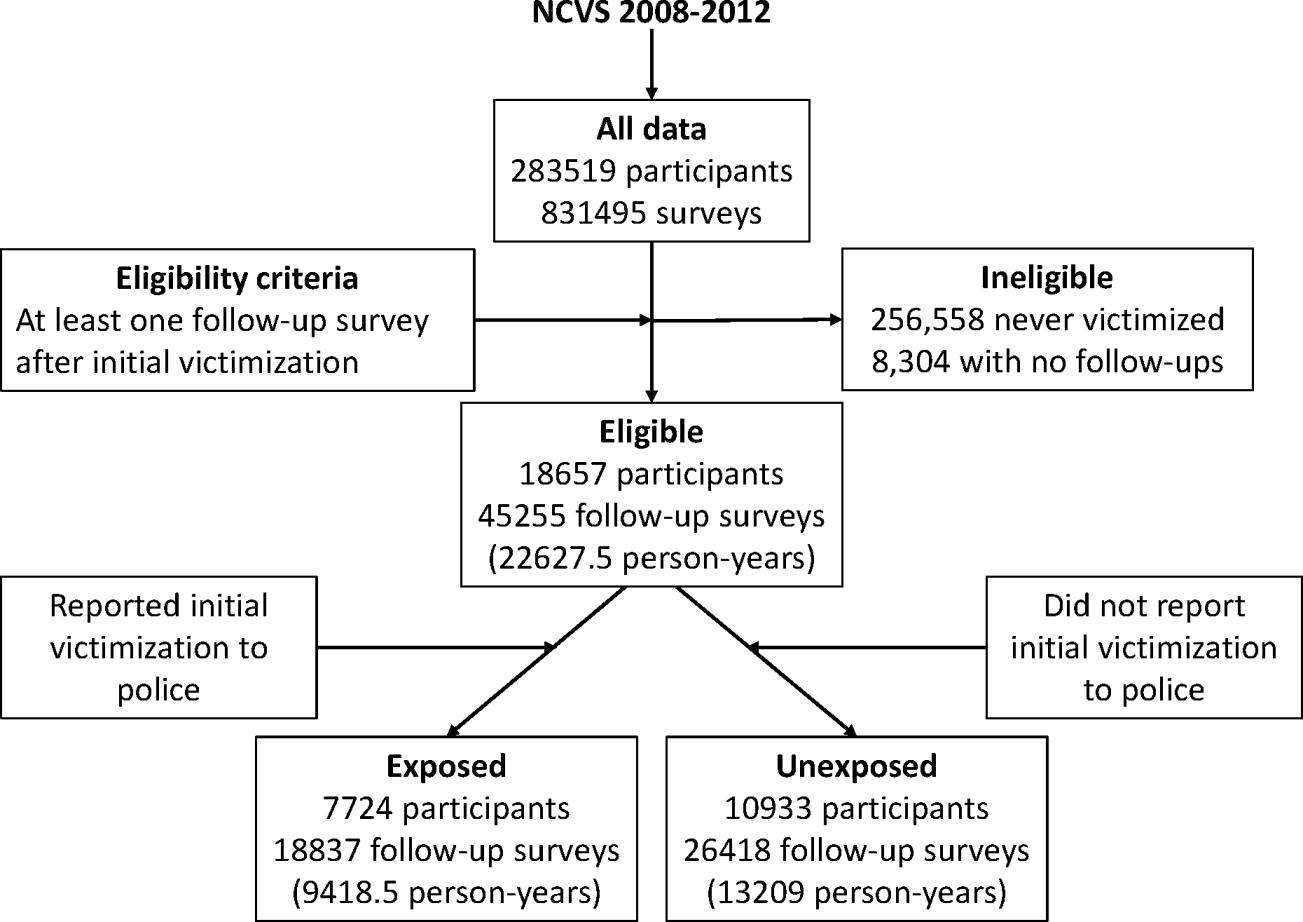
Participant selection and exposure distribution in the NCVS, 2008–2012, US.

Between 2008 and 2012, a total of 283,519 individuals participated in the NCVS. There were 256,558 participants who were never victimized and 8304 who did not take follow-up surveys after initial victimization; these participants were excluded from this study. The remaining 18,657 participants who took at least one follow-up survey after their initial victimization were eligible for this study (Fig 2). They took a total of 45,255 follow-up surveys (median = 2; range = 1–6) and reported 10,155 subsequent victimizations (median = 1; range = 0–10), and provided 22627.5 person-years of follow-up (median = 1 year; range = 0.5–3 years). The initial victimizations of 7,724 (41%) participants were reported to police, while those of 10,933 participants were not reported to the police. Of the 45,255 follow-up interviews, 899 were taken by proxies.

The median age of all eligible participants was 39.7 years (interquartile range: 26.2, 52.2), 50.4% of whom were females (Table 1). The majority of participants were White (78.3%), followed by African American (15.7%). At baseline, more than two thirds of the participants were victims of thefts, another 14.8% were victims of burglaries, and 16.3% were victims of interpersonal violence. The majority of offenders were males (80%); 14% of offenders were victim’s acquaintances. Most baseline victimizations (59.6%) occurred either inside or in the vicinity of victim’s home (outside).

**Table 1:** Baseline characteristics among those who reported to police and those who did not: NCVS 2008–2012, US.

Baseline Variables		Reported to police		Total (n = 18657)
		Yes (n = 7724)	No (n = 10933)	
**Victim**				
Age (years)	Median (IQR)	40.8 (28.8–52.7)	38.8 (24.1–52.0)	39.7 (26.2–52.2)
		**n (%)**	**n (%)**	**n (%)**
Sex	Female	4142 (51.6)	5656 (49.6)	9798 (50.4)
	Male	3582 (48.4)	5277 (50.4)	8859 (49.6)
Race	White	6178 (77.9)	8838 (78.7)	15016 (78.3)
	African American	1167 (17.0)	1414 (14.8)	2581 (15.7)
	AI/ AN	155 (2.1)	257 (2.4)	412 (2.2)
	A/ H/ PI	224 (3.0)	424 (4.1)	648 (3.7)
Ethnicity	Hispanic	1075 (14.1)	1797 (16.9)	2872 (15.8)
	Non-Hispanic	6649 (85.9)	9136 (83.1)	15785 (84.2)
Education	< HS	1483 (20.3)	2830 (28.3)	4313 (25.0)
	HS graduate	1962 (25.3)	2478 (22.4)	4440 (23.5)
	Some college	1707 (22.4)	2293 (20.8)	4000 (21.5)
	Associate	662 (8.3)	805 (7.0)	1467 (7.5)
	Bachelor	1327 (16.7)	1688 (14.6)	3015 (15.5)
	Master or higher	583 (7.1)	839 (6.9)	1422 (7.0)
Income^a^	< $25k	3534 (46.0)	4816 (44.2)	8350 (45.0)
	$25k - $49,999	1591 (20.4)	2340 (21.3)	3931 (20.9)
	$50k - $74,999	942 (12.2)	1363 (12.5)	2305 (12.4)
	$75k or more	1657 (21.4)	2414 (22.0)	4071 (21.7)
**Offender**				
Sex	Female	342 (4.6)	467 (4.4)	809 (4.5)
	Male	1613 (21.8)	1628 (15.6)	3241 (18.1)
	Not Known	5769 (73.6)	8838 (80.0)	14607 (77.4)
**V-O relation**	Acquaintance	1089 (14.6)	1354 (12.9)	2443 (13.6)
	Stranger	6635 (85.4)	9579 (87.1)	16214 (86.4)
**Location**^b^	Inside home	2419 (30.4)	2379(21.0)	4798 (24.8)
	Outside home	2552 (32.5)	4164 (36.5)	6716 (34.8)
	Friend's home	333 (4.7)	404 (4.1)	737 (4.3)
	Commercial place	410 (5.3)	757 (7.0)	1167 (6.3)
	Parking places	886 (11.6)	848 (7.7)	1734 (9.3)
	School	272 (4.1)	1019 (10.9)	1291 (8.1)
	Public places	530 (7.4)	725 (7.1)	1255 (7.2)
	Other	322 (4.1)	637 (5.7)	959 (5.1)
**Nature of baseline victimization**	Interpersonal violence	1488 (20.6)	1372 (13.4)	2860 (16.3)
	Burglary	1716 (21.4)	1187 (10.2)	2903 (14.8)
	Theft	4520 (58.0)	8374 (76.4)	12894 (68.9)

A/H/ PI, Asian/ Hawaiian/ Pacific Islander; AI/ AN, American Indian Alaska Native; HS, high school; IQR, interquartile range; V-O relation, victim offender relationship. n = unweighted frequency; % = weighted column percentage

^a^ annual family income in US dollars

^b^ location where the victimization took place.

The initial victimization was reported to the police more often by females (41.8%) than males (39.9%); by African Americans (44.2%) than Whites (40.6%), American Indians and Alaska natives (37.6%), or Asian, Hawaiians, or pacific islanders (33.7%); by non-Hispanics (41.6%) than Hispanics (36.7%); by those with less than $25,000 annual income (41.8%) than those with at least a $25,000 annual income (40.1%); in victimizations where the offender was an acquaintance (43.9%) than where the offender was a stranger (40.4%); by high school or more educated victims (59.1%) than victims with less than high school education (33.1%). The most often reported initial victimization was burglary (59.1%) followed by interpersonal violence (51.5%) and theft (34.4%). Initial victimizations that occurred in school were less often reported to the police (20.5%) than victimizations that occurred at home (49.9%), outside the home (38.1%), friend’s home (55.8%), commercial place (34.2%), parking places (51.1%), public places (41.8%), and other locations (33.3%).

The crude rate of all subsequent victimizations among those who reported their initial victimization to the police was 42.8/ 100 person-years (95%CI: 40.7, 44.8), compared to 55.0/ 100 person-years (95%CI: 53.0, 57.0) among those who did not report initial victimization to the police (Table 2). The adjusted rate difference of subsequent victimizations between exposed and unexposed was -13.5/ 100 person years (95%CI: -16.3, -10.7).

**Table 2:** Association of Police Reporting With the Incidence of Future Victimizations in the NCVS 2008–2012, US.

	Victimizations (n = 10,155)	Weighted unadjusted rates^a^ (95% CI)	Weighted adjusted^b^ exposed vs unexposed measures			
			RD	95% CI	RR	95% CI
**All victimizations**						
Reporting	3669	42.8 (40.7, 44.8)	-13.5	-16.3, -10.7	0.78	0.74, 0.82
Non-reporting	6486	55.0 (53.0, 57.0)	0		1	
**Interpersonal violence**						
Reporting	947	11.5 (10.5, 12.5)	-4.1	-5.5, -2.6	0.80	0.72, 0.90
Non-reporting	1604	14.5 (13.4, 15.5)	0		1	
**Burglaries**						
Reporting	824	9.2 (8.3, 10.1)	-0.5	-1.6, 0.6	0.95	0.84, 1.07
Non-reporting	942	7.3 (6.7, 7.9)	0		1	
**Thefts**						
Reporting	1898	22.1 (20.8, 23.4)	-8.9	-10.8, -7.0	0.73	0.68, 0.78
Non-reporting	3940	33.2 (31.8, 34.6)	0		1	

CI, confidence interval; RD, rate difference; RR, rate ratio

^a^ per 100 person-years

^b^ adjusted for baseline characteristics victim’s age, sex, race, income, education, offender sex, victim-offender relationship, victimization location, and the type of baseline victimization.

The adjusted RR suggest that overall future victimizations were 22% lower when the initial victimization was reported to the police relative to when it was not reported (RR: 0.78, 95%CI: 0.74, 0.82), future interpersonal violence victimizations were 20% lower (RR: 0.80, 95%CI: 0.72, 0.90), and future thefts were 27% lower (RR: 0.73, 95% CI: 0.68, 0.78) (Table 2). Future burglaries, however, did not decline with police reporting relative to not reporting (RR: 0.95, 95%CI: 0.84, 1.07) (Table 2).

The results from the sensitivity analysis show that the rate ratio for all future victimizations from the 6 month follow-up restricted (cutoff) sample was 0.76 (95% CI: 0.73, 0.80); from the 12 month follow-up restricted sample, RR = 0.77 (95% CI: 0.73, 0.80); and from the 24 month follow-up restricted sample, RR = 0.78 (95% CI: 0.74, 0.81). In essence, the rate ratios after sample restriction did not change substantively compared to the overall RR reported in Table 2, 0.78 (95% CI: 0.74, 0.82). This suggests that our results are robust and there may not be any selection bias arising due to the timing of initial victimization and the amount of follow-up.

Police reporting was associated with fewer subsequent victimizations among all racial/ ethnic groups as compared to not reporting (Table 3). We observed an effect measure modification of the exposure-outcome relationship by initial victim-offender relationship among the African Americans but not among other racial/ ethnic groups (Table 3). African Americans who reported stranger perpetrated victimization to the police did not experience a decline in the rate of future victimization as compared to African Americans who did not report to the police (RR:0.96, 95%CI: 0.83, 1.11).

**Table 3:** Adjusted^a^ police reporting vs non-reporting rate ratios by initial victim-offender relationship and victim’s race/ethnicity: NCVS 2008–2012, US.

Victim race/ ethnicity	Victim-Offender Relationship		Overall
	Acquaintance	Stranger	
**All victimizations**	0.71 (0.63, 0.80)	0.79 (0.75, 0.84)	0.78 (0.74, 0.82)
Whites	0.74 (0.65, 0.84)	0.77 (0.73, 0.82)	0.77 (0.72, 0.81)
African Americans	0.63 (0.47, 0.85)^b^	0.96 (0.83, 1.11)^b^	0.89 (0.8, 1.02)
American Indian/ Alaska Natives	0.81 (0.46, 1.43)	0.69 (0.52, 0.92)	0.71 (0.54, 0.92)
Asian/ Hawaiian/ Pacific Islander	0.72 (0.35, 1.46)	0.77 (0.54, 1.12)	0.77 (0.55, 1.06)
Hispanics	0.84 (0.60, 1.20)	0.78 (0.68, 0.90)	0.79 (0.69, 0.90)
Non-Hispanics	0.70 (0.61, 0.79)	0.79 (0.75, 0.84)	0.77 (0.73, 0.82)
**Interpersonal Violence**	0.76 (0.62, 0.93)	0.83 (0.71, 0.96)	0.80 (0.72, 0.90)
Whites	0.75 (0.61, 0.91)	0.82 (0.70, 0.95)	0.80 (0.71, 0.91)
African Americans	0.76 (0.51, 1.13)	0.86 (0.60, 1.23)	0.82 (0.63, 1.08)
American Indian/ Alaska Natives	1.25 (0.54, 2.89)	1.16 (0.63, 2.13)	1.18 (0.70, 2.00)
Asian/ Hawaiian/ Pacific Islander	0.79 (0.31, 2.00)	0.37 (0.14, 0.98)	0.48 (0.17, 1.30)
Hispanics	1.21 (0.75, 1.94)	0.89 (0.63, 1.24)	0.97 (0.73, 1.28)
Non-Hispanics	0.72 (0.60, 0.87)	0.81 (0.70, 0.94)	0.78 (0.69, 0.88)
**Theft**	0.64 (0.53, 0.77)	0.74 (0.69, 0.80)	0.73 (0.68, 0.78)
Whites	0.70 (0.57, 0.86)	0.72 (0.67, 0.78)	0.72 (0.67, 0.78)
African Americans	0.46 (0.26, 0.81)	0.93 (0.77, 1.11)	0.86 (0.72, 1.02)
American Indian/ Alaska Natives	0.97 (0.37, 2.55)	0.53 (0.35, 0.81)	0.59 (0.40, 0.87)
Asian/ Hawaiian/ Pacific Islander	-	0.82 (0.51, 1.30)	0.73 (0.48, 1.10)
Hispanics	0.50 (0.28, 0.90)	0.76 (0.64, 0.90)	0.74 (0.62, 0.87)
Non-Hispanics	0.66 (0.54, 0.81)	0.74 (0.68, 0.80)	0.73 (0.68, 0.78)
**Burglary**	0.92 (0.64, 1.33)	0.95 (0.84, 1.08)	0.95 (0.84, 1.07)
Whites	1.13 (0.74, 1.71)	0.92 (0.80, 1.06)	0.94 (0.82, 1.08)
African Americans	0.42 (0.14, 1.25)	1.21 (0.89, 1.63)	1.13 (0.85, 1.52)
American Indian/ Alaska Natives	0.77 (0.39, 1.52)	0.38 (0.08, 1.79)	0.70 (0.36, 1.35)
Asian/ Hawaiian/ Pacific Islander	1.26 (0.13, 12.26)	0.66 (0.30, 1.46)	0.70 (0.34, 1.44)
Hispanics	1.43 (0.45, 4.60)	0.75 (0.53, 1.06)	0.80 (0.57, 1.12)
Non-Hispanics	0.89 (0.60, 1.32)	0.98 (0.86, 1.13)	0.98 (0.86, 1.11)

^a^ adjusted for victim’s baseline age, sex, race, income, education, baseline victimization offender age, sex, gang affiliation, offender on drug or alcohol, victim-offender relationship for baseline victimization, and location and nature of baseline victimization

^b^ statistical significance for effect measure modification (*p-homogeneity* < 0.05).

Although insignificant due to small sample sizes, there were other race and initial victim-offender relationship based differences worth mentioning (Table 3). African Americans who reported stranger perpetrated victimization to the police experienced a higher rate of burglaries (RR: 1.21, 95%CI: 0.89, 1.63) and no change in rate of thefts (RR: 0.93, 95%CI: 0.77, 1.11) as compared to African Americans who did not report stranger-perpetrated victimization to the police. American and Alaska Natives who reported to the police experienced slightly higher subsequent interpersonal violence victimizations as compared to Natives who did not report to the police (RR: 1.18, 95%CI: 0.70, 2.00). Hispanics who reported to the police did not experience any change in the rate of subsequent interpersonal violence victimization as compared to the Hispanics who did not report (RR: 0.97, 95%CI: 0.73, 1.28). However, Hispanics who reported an acquaintance perpetrated victimization experienced more subsequent victimizations as compared to Hispanics who did not report acquaintance perpetrated interpersonal violence (RR: 1.21, 95%CI: 0.75, 1.94).

## Discussion

In this study, we used a nationally generalizable NCVS data, and observed that police reporting was associated with 22% fewer subsequent victimizations, particularly 20% fewer interpersonal violence victimizations and 27% fewer thefts. For every 100 victimizations that were reported to the police there were 13.5 fewer subsequent victimizations, four of which were interpersonal violence and nine of which were thefts. Overall, participants from all racial groups who reported to the police experienced fewer future victimizations.

Our results align with the study conducted in Cardiff, UK, where anonymized police reporting of violent crime indices from emergency department visits was associated with 42% fewer violence-related injuries in Cardiff [26]. However, this association is ecologic [26]. In this study, we evaluated the association of police reporting with the incidence of subsequent victimization on the same victims. The only other similar study was conducted among victims of intimate partner violence [25]. In that study, police involvement was associated with 48% fewer future incidences of domestic violence. This study also used the NCVS data to evaluate the repeat domestic violence victimizations from 1992–2002 [25].

There are several reasons to expect that police reporting of an incident might deter future victimization. First, police action may result in arrest and conviction of offenders thereby protecting the victim from re-victimization by the same offender. Second, the offenders may also view police intervention, irrespective of arrest, as stigmatizing, which may deter them from targeting the same victim again [30]. The stigma and shaming may be further compounded if/when police investigations acquire and release digital evidence (images or videos) that end up on social media and news outlets [31,32]. The digital evidence may also aid the police in identifying, arresting, and convicting the offenders [31]. Third, offenders may be deterred if the police warn or indicate future arrest is likely when they intervene [33]. However, the deterrence potential of stigma and arrest might be effective insofar as the same offender, whether acquaintance or stranger, intends to target that victim again [34]. Fourth, victims might acquire strategies to effectively lower their vulnerability to victimization as a result of their contact with the police. For instance, the police might raise their awareness about high-risk activities and neighborhoods and inform victims of strategies to safeguard themselves from future harm. Information and support is often provided by victim services that are generally affiliated with local police jurisdictions [35]. Lastly, there is evidence to suggest that reporting to police improves seeking of mental and physical health services by victims, which may also reduce the potential for subsequent victimization [35]. Altogether, these potential preventive outcomes of police notification may lower the risk of future victimizations as seen in the current study.

However, we observed that only 41% study participants reported their initial victimization to police. This is similar to previous studies that revealed the low prevalence of police reporting of victimizations [8–14]. Low reporting of victimization is also prevalent in cyber victimizations [36]. Several factors like retaliation from offenders, perceived triviality of the crime, relationship to the offender, societal stigma and stress, or distrust and perceived incompetence of law enforcement may explain the low prevalence of police reporting by victims [7,9].

While all other racial groups experienced 23% or more decline in subsequent victimization rates, African Americans experienced only an 11% decline. This was because in this study the African Americans who reported stranger perpetrated victimization experienced similar or higher rates of victimizations, especially property crimes (burglary), than African Americans who did not report stranger perpetrated victimization to the police (Table 3). This may suggest a lack of trust between the police and African American victims as suggested in previous studies from Philadelphia [16] and Washington, DC [17]. American Indians and Alaska Natives who reported to the police experienced a higher rate of acquaintance perpetrated interpersonal violence victimization (Table 3). American Indians and Alaska Native populations have been noted to experience high rates of violence victimizations [3], and the most common reason of not reporting violent victimizations to police is fear of reprisal [19] suggesting a potential retaliatory response.

### Limitations

In this study, we used self-reported NCVS data with 6 month recall, which may induce recall bias leading to under- or over-reporting of victimization to police and exposure. There are three possibilities. First, victims may under- or over-report future victimizations. If the under- or over-reporting is non-differential between the exposed and unexposed, then the rate ratio estimates will not be affected, but, the rate difference estimates will decrease or increase, respectively. The second possibility is under-reporting (more likely than over-reporting) of both exposed and unexposed events at the initial victimization (under-reporting of person-time), which will also result in under-reporting of future victimizations (under-reporting of outcomes); assuming non-differential reporting, this will not change the estimates. The third, and the most plausible, is differential reporting of the initial victimizations. Here, the exposed events are likely to be reported accurately–because police are usually informed in case of more traumatic events which may be recalled accurately; and, the unexposed are under-reported leading to under-reporting of future victimizations. This possibility will also not change any estimates. Hence, our estimates seem robust to recall bias.

About 1.9% (n = 899) of interviews were proxy interviews, which may be of concern, however, after excluding the proxy interviews from the analyses our estimates did not change appreciably.

Police reporting is not the same as police action or arrests. Our findings may be interpreted to suggest that police reporting may have a protective association with future victimization independent of police action; however, most police reporting is followed by some police action whether the victim is aware of it or not [26].

Lastly, although our estimates are generated from a well-grounded specification, this is an observational study. Even after controlling for many potential confounders there may be other unknown/unmeasured confounders that we cannot control for in the analyses. Hence, the rate ratios from this study are measures of association, not causation. Since we cannot conduct a randomized trial to observe this exposure-outcome relationship, the best potential way to get closer to a causal interpretation may be through a natural experiment or instrumental variable approach. Such an approach posits that if there is a randomly distributed natural variable (e.g., a policy that may be instituted in some states but not others) which predicts the exposure (prevalence of police reporting), does not have a direct relationship with the outcome (future victimization), and is not associated with known confounders of the exposure-outcome relationship, then we may be able to estimate the effect (causation) of the exposure on the outcome.

## Conclusions

We conducted a longitudinal study that allowed us to temporally examine how the rates of future victimization change following a police report for baseline victimization in a nationally representative sample of survey participants. Our results suggest that reporting victimizations to police may reduce the victim’s likelihood of future victimization. The protective association of police reporting may be due to the victim’s protective behaviors, police actions, and victim services that provide mental and physical health services. However, in this study, less than half of the victims reported their victimizations to the police. It is hence foreseeable, that an increase in reporting of violent and property crimes to police may be an important factor in crime prevention and control. Future studies may explore interventions to increase the prevalence of police reporting by victims thereby reducing re-victimizations in future.
